# Revealing the effects of *Aspergillus cristatus*, golden flower fungus, fermenting on the roots of the medicinal and edible plant *Panax ginseng*

**DOI:** 10.3389/fmicb.2026.1803757

**Published:** 2026-04-20

**Authors:** Lei Wang, Qi Wei, Chuhan He, Xu Li, Shouan Liu

**Affiliations:** 1 Institute of Chemical and Industrial Bioengineering, Jilin Engineering Normal University, Changchun, China; 2 Laboratory of Tea and Medicinal Plant Biology, College of Plant Sciences, Jilin University, Changchun, China; 3 Laboratory of Molecular Plant Pathology, College of Plant Sciences, Jilin University, Changchun, China

**Keywords:** *Aspergillus cristatus*, ginsenosides, metabolome, *Panax ginseng*, transcriptome, transformation

## Abstract

*Panax ginseng* is one of the most important medicinal and edible plants with pharmacological compounds mainly concentrated in the roots. Although chemical transformations of ginseng active compounds have been studied, their biotransformation processes by beneficial microbes are less reported. This study aimed to reveal changes in the functional components in *P. ginseng* roots fermented by the golden flower fungus *Aspergillus cristatus*. *P. ginseng* roots were incubated with *A. cristatus,* and the final product was given the name “Golden Flower Chinese Ginseng (GFCG).” A high-performance liquid chromatography (HP-LC) and liquid chromatograph mass spectrometer (LC–MS) system revealed that fermentation by *A. cristatus* caused metabolite changes in GFCG and promoted the production of rare ginsenosides. Transcriptomic analysis demonstrated that more than 72% of significantly differentially expressed genes in *A. cristatus* showed a decrease during interaction with *P. ginseng*. In the meantime, transcription-related genes were suppressed, while translational and post-translational events were activated, suggesting a special role of the fungal microbe when it is colonizing medical and edible plants. Therefore, this study provides detailed chemical characterization of GFCG and the potential molecular mechanism underlying the biotransformation of *P. ginseng.*

## 1 Introduction

*Panax ginseng* is one of the medicinal and edible plants that has been used in China for a long time in history, where it has been recognized as both a therapeutic agent and a dietary ingredient for millennia. It is widely cultivated and utilized across East Asian countries such as China, Korea, and Japan, and is officially monographed in the United States Pharmacopeia (USP) (Fan et al., 2019; Yun, 2001). Owing to over 100 functional bioactive components in *P. ginseng* roots, primarily encompassing saponins, volatile oil, polysaccharides, and amino acids, its bioactive constituents exhibit multiple health-promoting effects, including anti-aging, anti-tumor, anti-fatigue, anti-inflammatory, antioxidant, hypoglycemic effects, immunomodulatory effects, neuroprotective effects, cardioprotective effects, and metabolic regulatory effects (Jolly et al., 2024; Kim et al., 2017; Lee and Lee, 2024; Liu, 2012; Piao et al., 2020; Tong et al., 2022; Yao and Guan, 2022; Zhang et al., 2023). In addition, *P. ginseng* has been officially approved as both a dietary supplement and functional food in China, reflecting its established safety profile and nutritional value (Fan et al., 2019; Jin et al., 2015).

Among the active compounds in *P. ginseng*, saponins represent the most extensively studied and pharmacologically significant chemical class. To date, over 300 distinct ginsenosides have been isolated and structurally elucidated from various *Panax* species and their respective plant parts, including roots, leaves, stems, and flower buds (Hou et al., 2021). Generally, there are four main types of ginsenosides, which are structurally characterized according to different aglycones, which include protopanaxadiol (PPD)-type, protopanaxatriol (PPT)-type, oleanane-type, and ocotillol (OT)-type (Hou et al., 2021). The rare ginsenosides, which occur in trace amounts or are virtually absent in native plant tissues, are now reported to exhibit superior pharmacological potency compared to the major ginsenosides since their ease of absorption by the human body (Kim et al., 2019; Zheng et al., 2021). Beyond ginsenosides, non-ginsenoside components such as flavonoids, phenolic acids, and alkaloids are also reported to have significant pharmacological effects (Fan et al., 2021; Sun et al., 2023).

It is noted that although many research studies on *P. ginseng* bioactive constituents mainly focus on the composition and pharmacology, their contents and types vary across different processes. Consequently, substantial effort has been devoted to improving the quality of *P. ginseng* products through various methods, such as frying, steaming, acid treatment, and fermentation (Huang et al., 2019; Huang et al., 2023; Piao et al., 2020; Xie et al., 2012). Commercial sterilization, that is, 121 °C and 30 min, is a commonly used thermal process in the food industry (Barbhuiya et al., 2021). Recent metabolomic analyses have demonstrated that this commercial sterilization induces obvious alterations in 88 terpenoids, including 30 ginsenosides, which indicates the improvement of the function of ginsengs (Zhang et al., 2022).

Microbial biotransformation of medicinal plants by beneficial microbes has been widely reported to enhance bioactivity, diversify active compounds, and reduce toxicity of products (Pinela et al., 2020; Tian et al., 2021; Wu et al., 2022). Among these beneficial microbes in the transformation of natural resources, *Aspergillus cristatus*, commonly known as the golden flower fungus, is the dominant fungus during Chinese Fuzhuan brick tea (FBT) fermentation (Ge et al., 2016). Natural materials fermented with *A. cristatus* demonstrate various physiological activities, such as the antioxidant activities of rice Koji and the anti-aging activity of *P. notoginseng* (Lee et al., 2021a; Lee et al., 2021b). Thus, fermentation by *A. cristatus* can be an effective biotechnological approach for producing new bioactive materials. Therefore, it is vital to elucidate the changes in functional components in *P. ginseng* induced by a beneficial microbe such as *A. cristatus*.

In this study, we incubated *P. ginseng* with *A. cristatus* to produce GFCG, and then performed transcriptome analysis to identify the differentially expressed genes (DEGs) in the fungus. We further conducted untargeted metabolomic profiling to characterize differentially accumulated metabolism (DAM) in GFCG. This study will provide a comprehensive understanding of the metabolite transformation routes and the molecular mechanisms that govern the beneficial microbe-caused bioconversion of *P. ginseng*.

## 2 Materials and methods

### 2.1 Plants and fungi materials

Notably, 4-year-old *P. ginseng* roots were collected from Hushentang Co., Ltd. (Fusong, Jilin, China). Ginseng roots were cut into pieces and sterilized by autoclaving at 115 °C for 15 min using an autoclave machine, with minor modifications. The sterilized ginseng roots were placed in 500-mL glass bottles, with 100 g in each, for inoculation with the golden flower fungus. The spores of *A. cristatus* (1 × 10^6^/mL) were evenly sprayed onto the surface of sterilized *P. ginseng* roots. The inoculated samples were incubated at 28 °C for 7–10 days. After fermentation, the ginseng roots were dried at 80 °C for 4 h, ground into powder, and screened through a 40-mesh sieve. The resulting power was used for metabolite and ginsenoside measurements, respectively. The ginseng roots without fungal inoculation (sprayed with ddH_2_O) served as the control.

The golden flower fungus *A. cristatus* was originally isolated from Fuzhuan dark tea and grown on Potato Dextrose Agar (PDA) plates for 10 days. The fungal spores were collected and inoculated onto sterilized ginseng roots for fermentation. For RNA-sequencing, the fungi were collected from ginseng roots, frozen in liquid nitrogen, and stored at −80 °C (labeled as EcRS). The fungi without interaction with ginseng were used as the control (spores harvested from plates, labeled as EcCK). Three replicates were performed, respectively.

### 2.2 HP-LC analysis of ginsenosides with or without fungal treatment

Accurately weighed 0.5 g *P. ginseng* powder into the 25 mL EP centrifuge vial, followed by the addition of 15 mL 80% methanol (v/v). This solution was sonicated for 30 min and then kept undisturbed overnight. After that, two additional sonication cycles were performed (30 min each) with a 1 h interval between cycles. After the final sonication, the volume was adjusted to 25 mL with 80% methanol, and the mixture was allowed to stand overnight again. To obtain a final, clear sample for high-performance liquid chromatography (HP-LC) analysis, the supernatants were collected and filtered through a 0.22 μm filter membrane.

For HP-LC analysis of ginsenosides, the chromatographic separation conditions are set as follows. The column: ZORBAX Eclipse Plus C18 column (4.6 × 250 mm, 5 μm). The mobile phase consisted of water (A) and acetonitrile (B). The gradient elution was conducted as follows: 0–15 min, 19% B; 15–25 min, 19–29% B; 25–35 min, 29–40% B; 35–65 min, 40–100% B; 65–75 min, 100–19% B. The detection wavelength: 203 nm. The column temperature: 35 °C. The flow rate: 1.0 mL/min. The injection volume: 5 μL. The standard ginsenosides, such as Re, Rb1, Rd, Rg3, Rg2, and Rh2, were purchased from Shanghai Winherb Medical Science Co., Ltd.

### 2.3 Transcriptome analysis of differentially expressed genes in *A. cristatus* before or after interaction with *P. ginseng*

For RNA-sequencing of the golden flower fungus *A. cristatus*, the following procedures were, respectively, performed as previously indicated (Chen et al., 2022; Liu et al., 2023) (i) total RNA identification, (ii) purification, (iii) monitoring, (iv) the cDNA library construction, (v) sequencing, (vi) the raw data cleaning, and (vii) the raw data analysis. In brief, clean reads were observed by first removing the lower quality reads from the raw data, including those containing adaptors, primers, and nucleotides with Q quality scores below the threshold. The Q20, Q30, and guanine or cytosine (GC) content of the clean data were calculated. All the above clean, high-quality data were used for downstream analysis.

To map fragments to the genome and quantify gene-level expression, an index of the fungal genome reference was built. Paired-end clean reads were aligned to the reference, and the fragments per kilobase of transcript per million mapped reads (FPKM) of each gene were then calculated. The *A. cristatus* reference genome and fungal genes annotation files were obtained from the NCBI genome datasets with assembly number ASM4470619v1 (Ge et al., 2016).

For analysis of differentially expressed genes (DEGs), DEGs in all samples (EcRS, EcCK) were analyzed as described in Chen et al. (2022). The DEGs were selected with log2 (fold change) ≥ 1 or log2 (fold change) ≤ 1 and with statistical significance (*p* value ≤ 0.05) by the R package.

### 2.4 Metabolome analysis of differentially accumulated compounds during *P. ginseng* roots infected by *A. cristatus*

For metabolite extraction from *P. ginseng* roots, samples were prepared as follows: *A. cristatus*-treated *P. ginseng* roots (RJ) and untreated ginseng roots (RCK) were extracted with 120 μL of precooled 50% methanol. The mix was vortexed for about 1 min, then incubated at room temperature for 10 min, and next centrifuged at 4,000 *g* for 20 min, and finally transferred the supernatants into a new 96-well plate. The quality-control (QC) samples (from pooled quality control) were also included by combining 10 μL of each extraction mixture.

The root samples were analyzed using the liquid chromatograph mass spectrometer system (LC–MS) according to the machine’s instructions from LC-Bio Technology Co., Ltd., Hangzhou, China. All chromatographic separations were done by an UltiMate 3,000 UP-LC system (Thermo Fisher Scientific, Bremen, Germany). The ACQUITY UP-LC T3 column (100 mm × 2.1 mm, 1.8 μm, Waters, Milford, CT, USA) was used for the reversed-phase separation. The column oven was kept at 40 °C.

The high-resolution tandem mass spectrometer Q-Exactive (Thermo Fisher Scientific, Bremen, Germany) was used to detect metabolites eluted from the column. The Q-Exactive was operated in positive and negative ion modes, respectively. Precursor spectra (70–1,050 m/z) were collected at 70,000 resolution to hit an AGC target of 3 × 10^6^. The maximum injection time was set to 100 ms. A top-3 configuration for data acquisition was set in DDA mode. Fragment spectra were collected at 17,500 resolution to hit an AGC target of 1 × 10^5^ with a maximum inject time of 80 ms.

After that, the LC–MS data were analyzed as follows: (i) transforming into the mzXML format by MSConvert, (ii) processing by the XCMS, CAMERA, and metaX toolbox, and (iii) implementing in the R software. The combined retention time (RT) and m/z data were used to identify each ion (Chen et al., 2022; Liu et al., 2023).

### 2.5 GO/KEGG analysis

Gene Ontology (GO) enrichment analysis of DEGs was implemented by the Goseq R package, in which gene length bias was corrected. Kyoto Encyclopedia of Genes and Genomes (KEGG) is a database resource based on large-scale molecular datasets generated by genome sequencing or metabolomics technologies. GO/KEGG analysis was performed as previously indicated (Chen et al., 2022; Liu et al., 2023).

### 2.6 Statistical analysis

Analysis of variance was performed using the Statistical Product and Service Solutions software (IBM). The results were considered significant as follows: *p < 0.05, **p < 0.01, ***p < 0.001. All data are represented as the mean ± standard error of the mean (SEM) of at least three replicates.

## 3 Results

### 3.1 Fermentation of golden flower fungus *A. cristatus* on the *P. ginseng* roots

**Figure 1:**
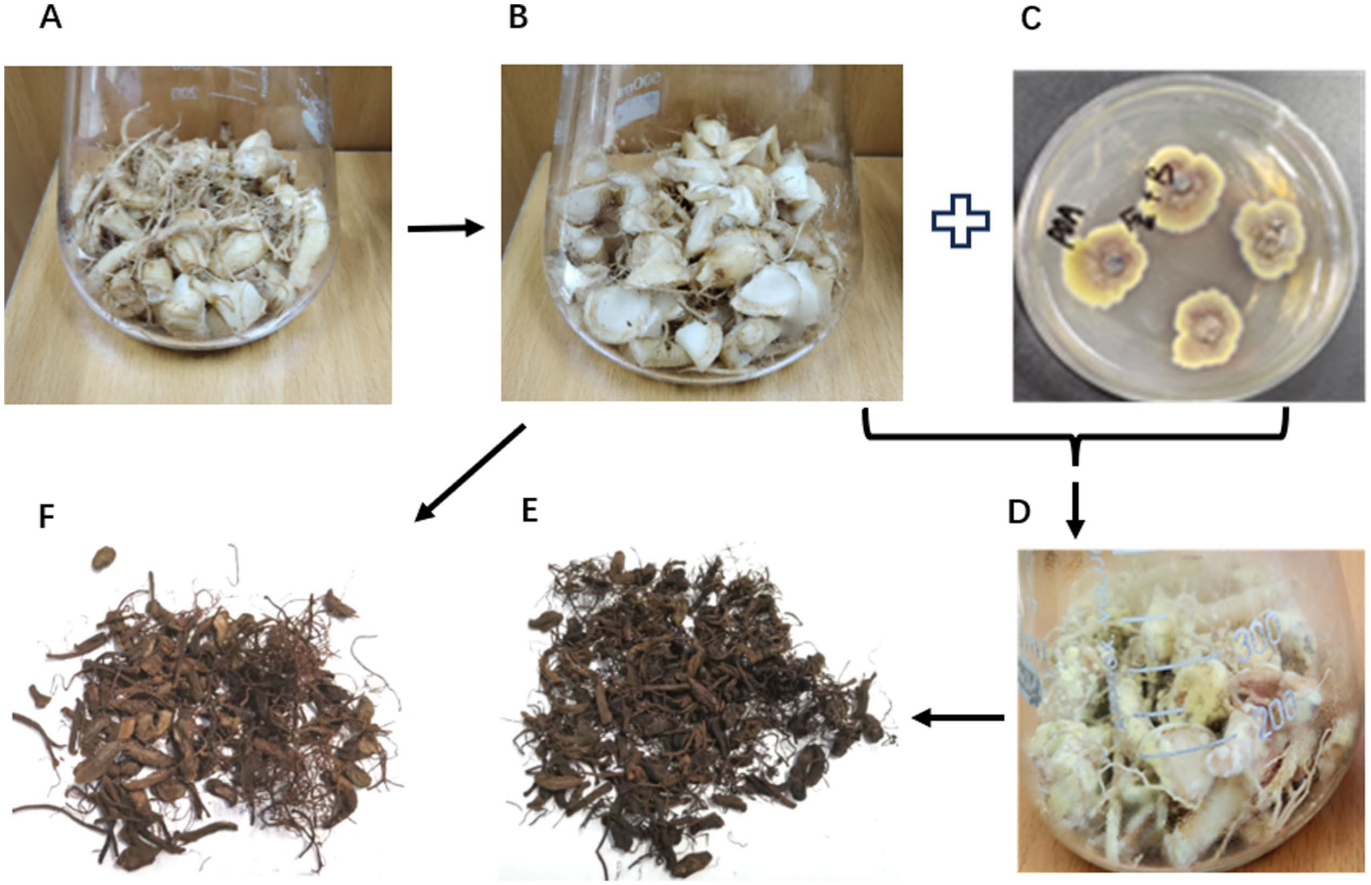
Schematic of the production process and characteristics of the *Aspergillus cristatus* fermented *Panax ginseng*. **(B–F)** The production process of GFCG and the CK.

To ferment with the golden flower fungus *A. cristatus*, *P. ginseng* roots were cut into pieces (Figure 1A). Since the field-grown *P. ginseng* roots may contain endogenous or rhizosphere microbes that could contaminate the golden flower fungus fermentation, sterilization was performed as a pretreatment step. In addition, to minimize thermal destruction of fresh ginseng compounds by the traditional commercial sterilization condition (121 °C, 30 min), the sterilization was carried out at 115 °C for 15 min (Figure 1B). Fermentation was performed by inoculating *A. cristatus* spores into each bottle and incubating for more than one week (Figures 1C,D). Visually, the fermented samples would show dense mycelial coverage. After 7 days, the fungi were fully grown on the surface of *P. ginseng* roots with golden flowers, and all fermented samples were covered with dense mycelium (Figure 1D). It indicated that *A. cristatus* successfully grew on the roots of *P. ginseng.* After fermentation, the samples were dried at 80 °C for 4 h. Thus, we finally observed the *A. cristatus*-treated samples (RJ, Figure 1E) and untreated samples (*P. ginseng* CK, RCK, Figure 1F). As indicated in Figures 1E,F, the color of dried ginseng CK was red-brown with light, while the color of *A. cristatus*-treated *P. ginseng* was deep, dark brown with less light. These results confirmed the successful preparation of “Golden Flower Chinese Ginseng (GFCG).” Since the fungal fermentation may influence *P. ginseng* quality, which would be very interesting, we did further investigation.

### 3.2 Effects of golden flower fungus *A. cristatus* fermentation on the saponins biotransformation in *P. ginseng* roots

**Figure 2:**
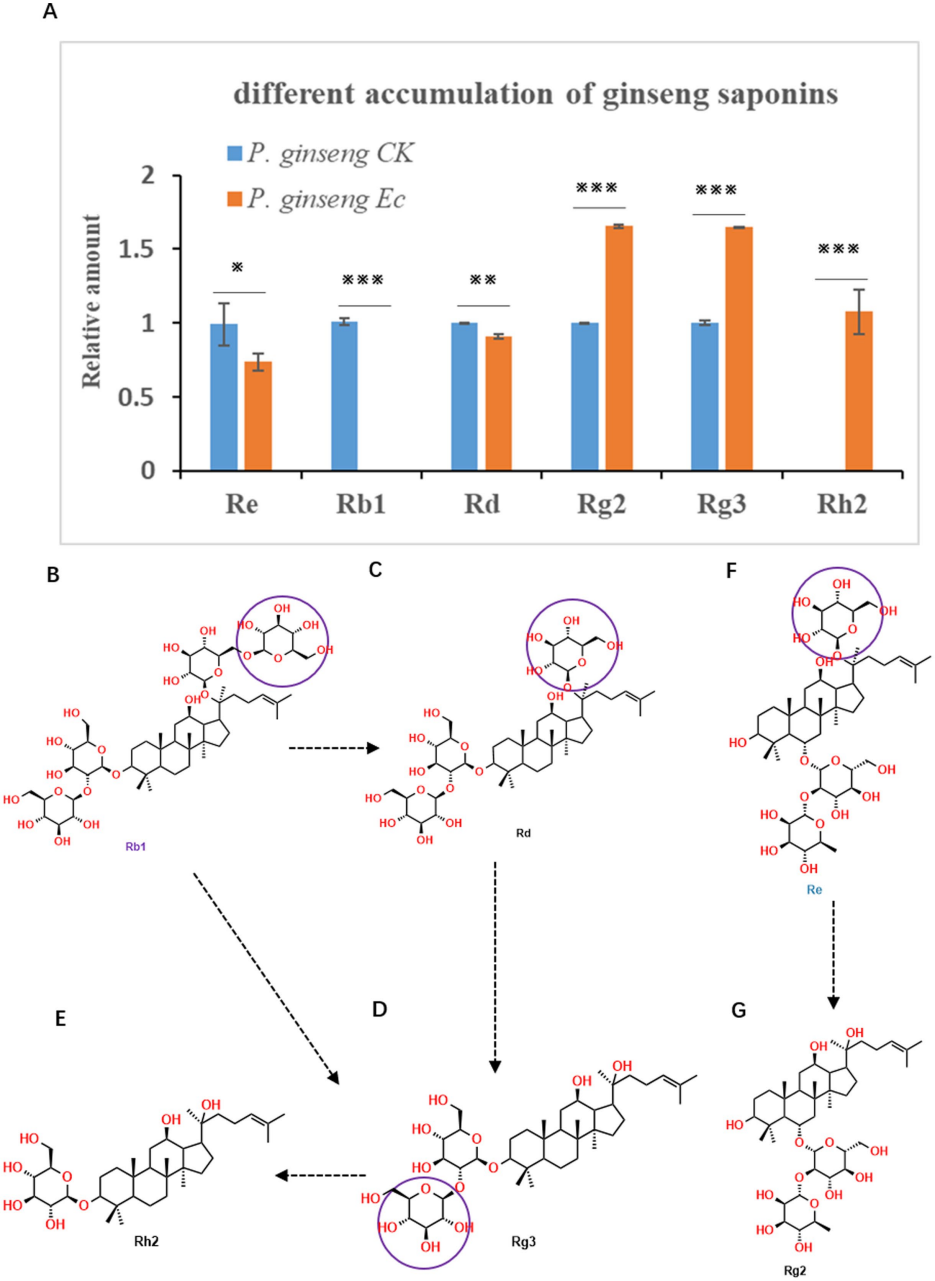
*Aspergillus cristatus* fermentation changed *Panax ginseng* saponins. **(A)** Differentially accumulated ginseng saponins in *P. ginseng* upon *A. cristatus* treatment and the untreated control samples (*p < 0.05; **p < 0.01; ***p < 0.001). **(B–G)** Structures of indicated ginseng saponins.

To evaluate whether *A. cristatus* fermentation could improve *Panax* quality, we measured and compared selected ginsenosides, which are pharmacologically important in *P. ginseng*. The samples of CK (*P. ginseng* CK) and the samples of *A. cristatus*-treated (*P. ginseng* Ec) were first ground into powder and extracted for HP-LC analysis. For the standards, the HP-LC grade of ginsenoside compounds was used. As indicated in Figure 2A, upon *A. cristatus* treatment, *P. ginseng* accumulated more ginsenosides Rg2 and Rg3 than the control (*p* < 0.001), while the accumulation of ginsenosides Re and Rd was significantly decreased (*p* < 0.05 and *p* < 0.01, respectively). Interestingly, the accumulation of Rb1 had almost disappeared in *P. ginseng* after *A. cristatus* treatment. On the contrary, the accumulation of ginsenoside Rh2 occurred only after *A. cristatus* treatment. These findings indicate the saponins could be biotransformed by the golden flower fungus *A. cristatus*.

Structural analysis of these ginsenosides provides mechanistic insights into the observed metabolic shifts. Based on the chemical structures of Rb1, Rd, Rg3, and Rh2, each compound lost one glucoside at a similar position (Figures 2B–E). Hereupon, in *A. cristatus* fermentation, we observed the decreasing concentrations of Rb1 and Rd, while increasing concentrations of Rg3 and Rh2, suggesting a biotransformation pathway could be Rb1 → Rd → Rg3 → Rh2, or from Rb1 → Rg3 → Rh2. Thus, the *P. ginseng* roots may induce the golden flower fungus *A. cristatus* (Figures 2F, G) to undergo transcriptional reprogramming and to induce the biosynthesis of certain enzymes that catalyze chemical transformations. Similarly, it may also happen from Re to Rg2 by enzymes from *A. cristatus.* Since ginseng ginsenosides, such as the Rg2, Rg3, and Rh2, are rare saponins that play key roles in medicine, the golden flower fungus *A. cristatus* fermentation represents an effective strategy for enhancing the medicinal value of *P. ginseng* via targeted biotransformation of bioactive compounds.

### 3.3 Transcriptome analysis of golden flower fungus *A. cristatus* during interaction with *P. ginseng* roots

To investigate the molecular mechanisms of *A. cristatus* during fermentation of *P. ginseng* and to reveal which genes are involved in the transformation of ginseng compounds, we performed RNA-sequencing after *A. cristatus* incubated fresh ginseng roots. One week post-incubation, the fungus grew, and the golden flower developed on the surface of the roots of fresh ginseng. Samples of *A. cristatus* were collected from the fungal-inoculated ginseng roots (EcRS). For the control, fungi without interaction with ginseng roots were collected (control, EcCK). After sequencing, around 286 million reads with high quality and about 7.15 G base pairs on average were generated in each library; 252 million validated high-quality reads were finally obtained (Supplementary Table S1). The raw sequence data have been submitted to the NCBI Short Read Archive under accession number GSE302790 (EcRS/EcCK). The reads were aligned to the *A. cristatus* genome (Ge et al., 2016).

**Figure 3:**
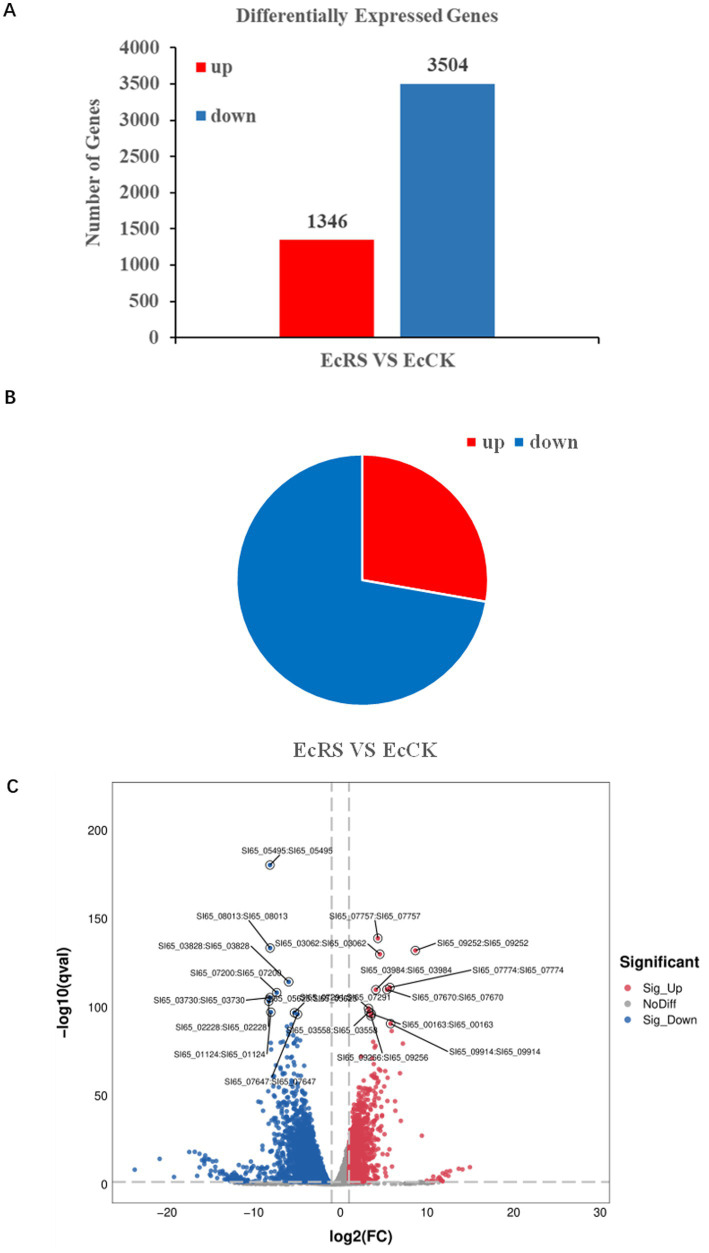
Differentially expressed genes (DEGs) in *Aspergillus cristatus* during fermenting *Panax ginseng*. **(A)** Numbers of significantly differentially expressed genes (≥ 2-fold; *p* ≤ 0.05) in *A. cristatus* after one week of interaction with *P. ginseng* roots by RNA-seq. **(B)** Percentage of up-regulated and down-regulated genes in *A. cristatus* upon interaction with *P. ginseng* (in total 4,850). **(C)** Volcano plot of differentially expressed genes with the labeled top genes of up-regulated or down-regulated genes.

To identify genes involved in *A. cristatus* after inoculation with *P. ginseng*, we compared statistically significantly differentially changed genes (altered 2-fold or more, *p* ≤ 0.05, sequence-specific transcription factors [SSTFs]) between the *A. cristatus* and *P. ginseng* interaction (EcRS) and the control (EcCK) (Figures 3A,C). 4,850 SSTF genes were identified in total compared with non-infected plants, with 1,346 genes being up-regulated and 3,504 genes being down-regulated (Figure 3A). Around 72.2% of SSTFs are down-regulated in *A. cristatus* during interaction with *P. ginseng* (Figure 3B). It indicates that many fungal genes (more than two-thirds) were repressed during interaction with *P. ginseng*.

As shown in Figure 3C, several top genes are up- or down-regulated in SSTF. Among up-regulated genes, *SI65_07291* encodes 1,3-beta-glucanosyltransferase; *SI65_03558* encodes beta-1,6-glucan biosynthesis protein; *SI65_03984* encodes lytic polysaccharide monooxygenase; *SI65_00163* encodes xylose isomerase-like protein; *SI65_07774* encodes protein involves in glucan catabolic process; *SI65_07757* encodes eukaryotic cysteine-rich secretory proteins, antigen 5, and pathogenesis-related 1 proteins (CAP) domain containing proteins; while *SI65_09252* encodes protein with Cyanovirin-N homologs (CVNHs) domain, CVNHs are a class of lectins known for their high-mannose sugar binding capabilities and antiviral properties. The up-regulation of these genes suggests that they may be involved in the biosynthesis of ginseng chemical metabolites, such as the conversion of rare ginsenoside compounds.

The down-regulated genes in *A. cristatus* after inoculation with *P. ginseng* included: *SI65_01124* encodes a putative RNA polymerase II transcription elongation factor; *SI65_07200* encodes a CHY zinc finger protein; *SI65_02228* encodes a pH-response regulator protein; *SI65_03730* encodes a protein belonging to the ABC transporter G family; while *SI65_07647* and *SI65_05495* encode agmatine deiminase family protein. The down-regulation of these genes suggests that they may play a role in the differential regulation of gene transcription, environmental sensing, and metabolic adaptation during the fungal transition from saprophytic to host-associated growth.

### 3.4 Transcriptional events changed to translational events during golden flower fungus–ginseng interaction

**Figure 4:**
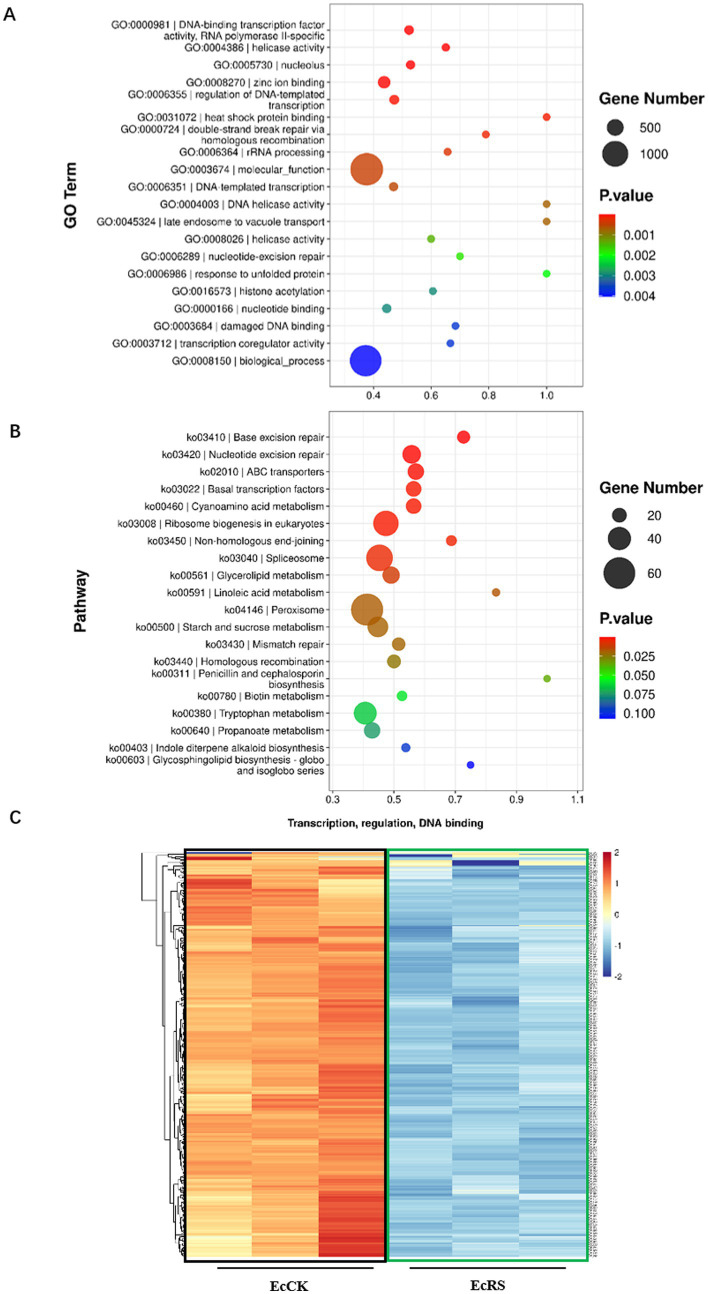
Gene enrichment analysis of the down-regulated SSTF genes in *Aspergillus cristatus*. **(A)** GO analysis of down-regulated genes in *A. cristatus*. **(B)** KEGG analysis of down-regulated genes in *A. cristatus*. **(C)** Heatmap analysis of DEGs in *A. cristatus* associated with transcription events.

**Figure 5:**
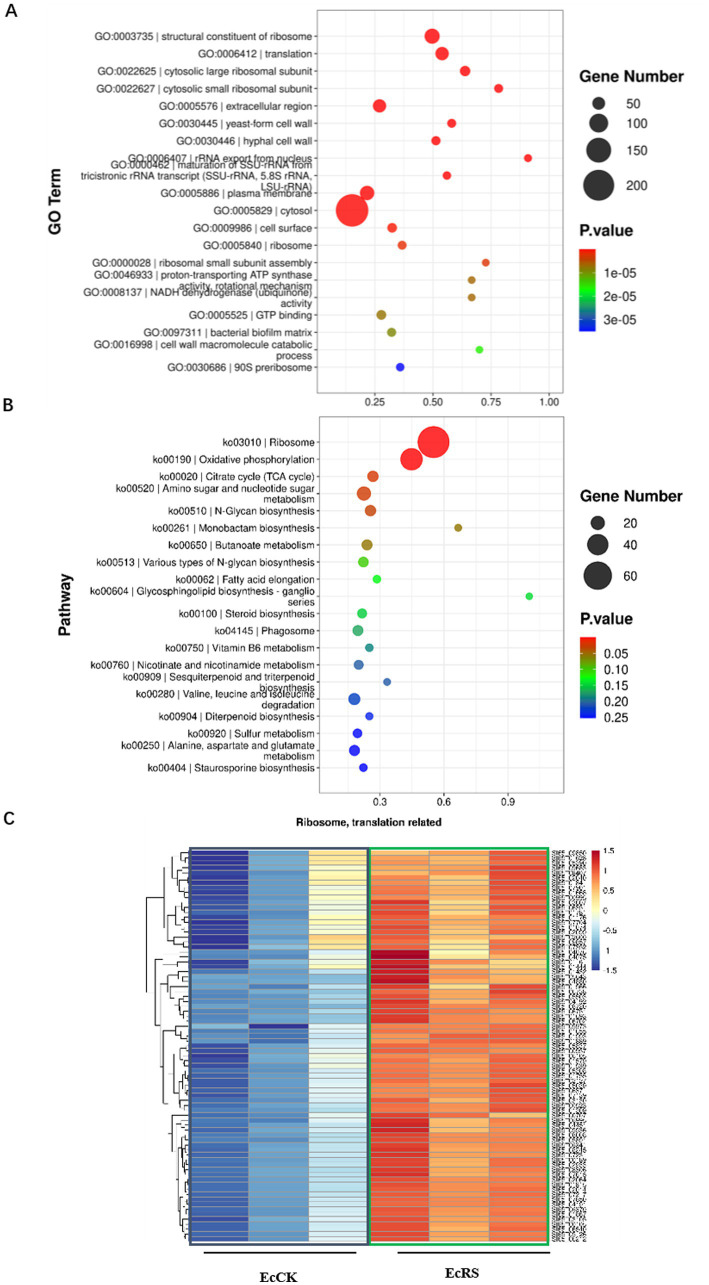
Gene enrichment analysis of the up-regulated SSTF genes in *Aspergillus cristatus*. **(A)** GO analysis of up-regulated genes in *A. cristatus*. **(B)** KEGG analysis of up-regulated genes in *A. cristatus*. **(C)** Heatmap analysis of DEGs in *A. cristatus* associated with translation events.

To systematically characterize the biological processes and pathways involved in the *A. cristatus* and *P. ginseng* interaction, we further analyzed up- and down-regulated genes using GO and KEGG methods, respectively (Figures 4, 5).

In the GO enrichment analysis, genes that play important roles in transcriptional regulation and DNA repair are enriched among down-regulated genes (Figure 4A; Supplementary material S1). These GO terms include DNA-binding transcription factor activity (RNA polymerase II-specific), regulation of DNA-templated transcription, DNA-templated transcription, transcription coregulator activity, helicase activity, DNA helicase activity, double-strand break repair via homologous recombination, damaged DNA binding, etc. However, many genes associated with translation, ribosome-related (structural constituent of ribosome, cytosolic large ribosomal subunit, cytosolic small ribosomal subunit, ribosome, ribosomal small subunit assembly), ribosomal RNA (rRNA) export from nucleus, maturation of small subunit ribosomal RNA SSU-rRNA from tricistronic rRNA transcript, plasma membrane, etc., are enriched in up-regulated genes (Figure 5A; Supplementary material S2).

For the KEGG pathway analysis, the genes associated with repair events (for example, the base excision repair, nucleotide excision repair, mismatch repair), basal transcription factor, ABC transporter, metabolism (cyanoamino acid metabolism, glycerolipid metabolism, linoleic acid metabolism), etc., are down-regulated (Figure 4B; Supplementary material S1), while genes associated with ribosome, amino sugar and nucleotide sugar metabolism, oxidative phosphorylation, N-glucan biosynthesis, etc., are enriched in up-regulated genes (Figure 5B; Supplementary material S2).

The GO and KEGG analyses of up- or down-regulated genes indicated that translation, transcription, DNA repair events, and metabolism-related genes are enriched in the golden flower fungus *A. cristatus* when interacting with the medicinal plant *P. ginseng*. While the transcription-related events were repressed (Figure 4C), the translation-related events were activated (Figure 5C), suggesting *P. ginseng* might interfere with fungal genes at the transcription level, while triggering changes in the fungal genes at the translational and post-translational levels.

### 3.5 Metabolomic analysis of the *P. ginseng* roots upon the golden flower fungus *A. cristatus* fermentation

Since *A. cristatus* changed its transcripts, particularly those related to fungal translation, which may be involved in *P. ginseng* root metabolism, a metabolome approach was then conducted using LC–MS. The objectives were to identify active compounds and to examine changes in the metabolites in *P. ginseng* following fermentation with *A. cristatus* (RJ) compared with untreated samples (RCK). The identified metabolites were assigned to the KEGG databases. Several top-ranking KEGG pathways were found to be significantly enriched (Supplementary Figure S1, KEGG pathway 3). The top 1 KEGG pathway was associated with metabolic pathways, while glycerophospholipid metabolism and the biosynthesis of secondary metabolites were the second- and third-most enriched KEGG pathways. Principal component analysis (PCA) and PLS-DA were performed to get a deep overview of the metabolic changes (Supplementary Figure S2). In the PCA score plot of ginseng based on all changed compounds, PC1 and PC2 accounted for 84.19 and 14.69%, respectively. In the score plot of PCA, the RCK samples were well clustered and were clearly distinguished from the RJ samples, indicating that the compounds in RJ remarkably changed. In the PLS-DA models, RCK and RJ were prominently separated.

**Figure 6:**
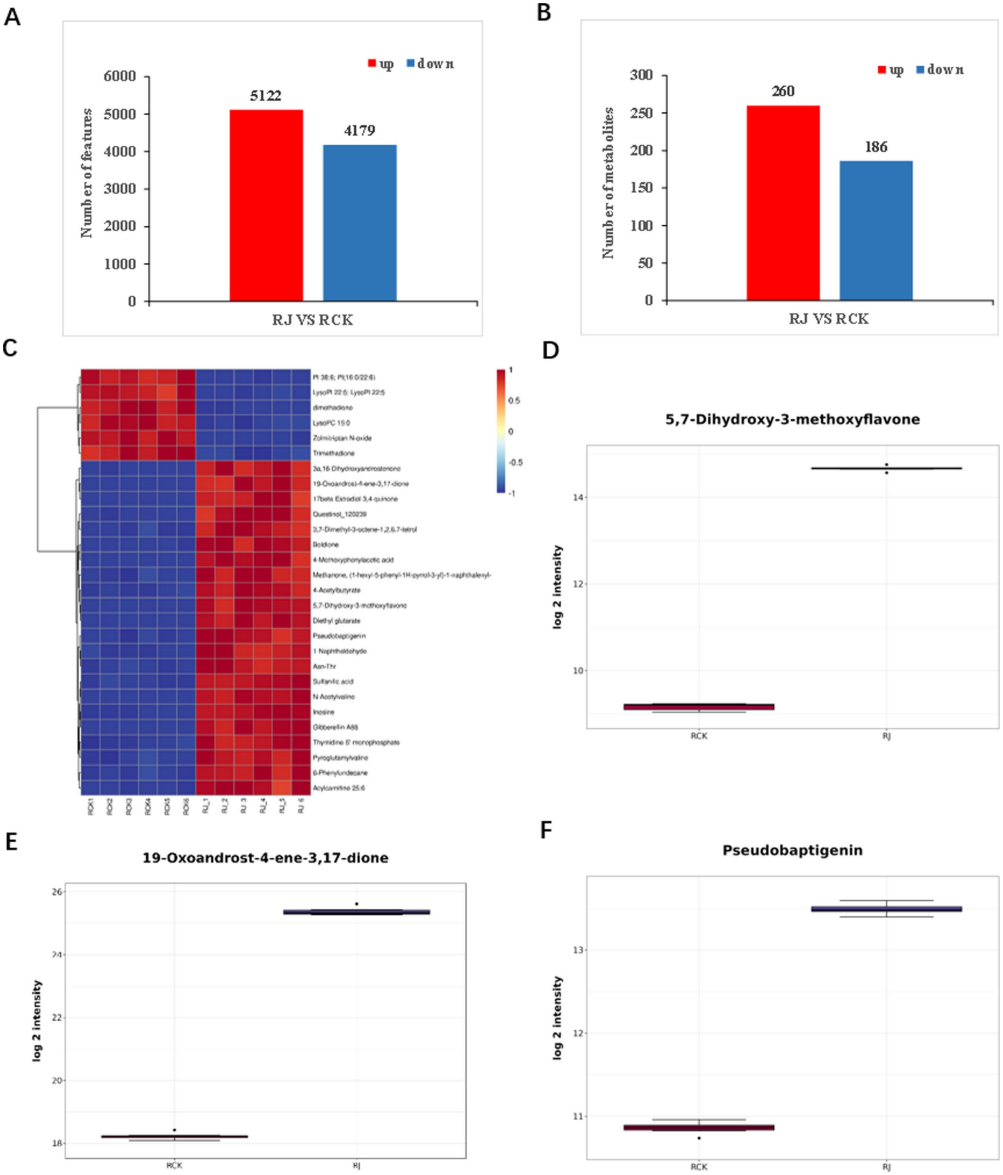
Differentially accumulated metabolites (DAMs) in *Panax ginseng* during interaction with *Aspergillus cristatus*. **(A)** The number of differentially changed features in *A. cristatus* treated *P. ginseng* (RJ) and the untreated control (RCK). **(B)** Numbers of significantly differentially changed metabolites in *A. cristatus* treated *P. ginseng* (RJ) and the untreated control (RCK). **(C)** Heatmap analysis of the top 28 differentially accumulated metabolites. **(D–F)** Differentially accumulated compounds in RCK and *A. cristatus*-treated *P. ginseng* (RJ).

The quantification finally identified 9,301 features in *P. ginseng*, with 5,122 of them being increased upon *A. cristatus* treatment compared with CK, while 4,179 of them were decreased (Figure 6A). 942 high-quality MS2 metabolites were used for differential analysis (Supplementary material S3; Supplementary Figure S3). The statistical analysis identified 446 significant differentially accumulated metabolites (DAMs) (Supplementary Figure S3). In the comparison between *A. cristatus* -infected *P. ginseng* and CK, 260 metabolites were up-regulated, and 186 were down-regulated (Figure 6B; Supplementary material S3; Supplementary Figure S3).

All DAMs were assigned to various metabolic categories. Supplementary Figure S4 shows the significantly enriched metabolic pathways observed by KEGG analysis. These include glycerophospholipid metabolism, glycerolipid metabolism, D-amino acid metabolism, biosynthesis of phenylpropanoids, fat digestion and absorption, vitamin digestion and absorption, biosynthesis of amino acids, steroid hormone biosynthesis, ABC transporters, glycosylphosphatidylinositol (GPI)–anchor biosynthesis, etc. These results suggest that inoculating *P. ginseng* with *A. cristatus* altered metabolites in the indicated pathways. For example, 398 metabolites related to lipid and lipid-like molecules were enriched upon *A. cristatus* incubation, and two-thirds of them were decreased (Supplementary Figure S5). 167 metabolites are associated with benzenoids, phenylpropanoids, and polyketides, while half of them are increased upon *A. cristatus* treatment (Supplementary Figure S6).

### 3.6 . Metabolites associated with food and pharmacological quality were enriched in ginseng roots upon fungal treatment

We next analyzed the top 28 differentially accumulated metabolites (DAMs) in *P. ginseng* with or without *A. cristatus* treatment. Heatmap analysis clearly indicated the DAMs between RJ and RCK (Figure 6C). Among these, several metabolites related to pharmacological functions were increased during *P. ginseng* and *A. cristatus* interaction (Figure 6C). These include 5,7-dihydroxy-3-methoxyflavone, 19-oxoandrost-4-ene-3,17-dione, pseudobaptigenin, zolmitriptan N-oxide, inosine, questinol, boldione, 17beta-estradiol-3,4-quinone, etc. (Figures 6C–F; Supplementary Figure S7). The induction of these compounds suggests that *A. cristatus* enhances the pharmacological effects of *P. ginseng*. The 5,7-dihydroxy-4′-methoxyflavone, also named acacetin, acts as a natural flavone found in various plants. Multiple studies have indicated that acacetin can be applied as a therapeutic and nutraceutical agent, possessing numerous pharmacological properties, including anti-cancer, anti-oxidant, anti-inflammatory, anti-diabetic, anti-infectious, and anti-obesity activities as well as cardiovascular protective, and neuroprotective (Semwal et al., 2019). 19-Oxoandrost-4-ene-3,17-dione is involved in the steroid hormone biosynthesis pathway and acts as a metabolite to distinguish Alzheimer’s disease participants from normal controls (Xi et al., 2021). Pseudobaptigenin has recently gained attention as a novel therapeutic agent for breast cancer, which is one of the most prevalent cancers and a major cause of death for women in the world. Hormone receptor therapies constitute a major strategy for breast cancer treatment. Pseudobaptigenin was identified as a potent antagonist of multiple hormone receptors, with the potential to inhibit breast cancer cell receptors (Ray et al., 2024). The induction of these metabolites further suggested that the golden flower fungus *A. cristatus* fermentation improved the bioactive compounds of *P. ginseng*.

### 3.7 Integrated transcriptomic and metabolomic analysis of golden flower fungus *A. cristatus* fermentation on *P. ginseng*

To investigate the probable association between genes and metabolites involved in a similar biological process, as well as the transduction pathway of ginseng metabolites during fungal fermentation, a comprehensive analysis of transcriptome and metabolome was performed using Pearson’s Correlation Coefficient as previously described (Chen et al., 2022). When comparing the transcriptomic changes of *A. cristatus* before and after fermentation with *P. ginseng*, as well as the metabolite changes in *P. ginseng* before and after fermentation by *A. cristatus*, our results indicated 4,850 SSTF genes participated in 120 pathways, while 446 differentially accumulated metabolites were involved in 184 pathways (Supplementary material S4).

The results of the comprehensive analysis between transcriptome and metabolome are shown in Supplementary Figure S8A and Supplementary material S5. A total of 53 common KEGG pathways were identified in both transcriptomic and metabolomic analyses. Among the differentially expressed genes (DEGs), ABC transporters, cyanoamino acid metabolism, and starch and sucrose metabolism were significantly enriched in common KEGG pathways. Among the differentially accumulated metabolites (DAMs), 15 pathways were significantly enriched. These include ABC transporters, arginine biosynthesis, arginine and proline metabolism, aminoacyl-tRNA biosynthesis, autophagy, biosynthesis of unsaturated fatty acids, fatty acid biosynthesis, glycerophospholipid metabolism, glycosylphosphatidylinositol (GPI)-anchor biosynthesis, glycerolipid metabolism, alpha-linolenic acid metabolism, phenylalanine metabolism, purine metabolism, tyrosine metabolism, and tryptophan metabolism.

Notably, the ABC transporters were significantly enriched both in transcriptome and metabolomic analysis (Supplementary Figure S8A; Supplementary material S5). In the ABC transporter pathway, the accumulation of sucrose, inosine, and glutamine increased in *P. ginseng* upon *A. cristatus* fermentation, while the accumulation of choline, arginine, adenosine, uridine, and trehalose decreased. For the transcripts, changes in the ABC transporter pathway during *A. cristatus* fermenting *P. ginseng*, most of the genes were decreased (Supplementary Figure S8A; Supplementary material S5). It indicated that many ABC transporter-related genes in *A. cristatus* were suppressed by *P. ginseng*, which might contribute to the differential accumulation of metabolites such as sucrose, inosine, glutamine, choline, arginine, adenosine, uridine, and trehalose. However, we only observed that the ABC transporter was significantly enriched in both transcriptome and metabolomic analysis.

## 4 Discussion

Chinese ginseng, scientifically named as *P. ginseng*, with a long medicinal history in China and used as one of the most valued traditional medicines, has gained increasing applications in the food industry. The enhancement of *P. ginseng*’s bioactive compounds attracted our interest. Traditional commercial sterilization is a typical thermal food process in the industry. When foods were sterilized by high-pressure steam, the primary effects were changes in nutritional values and the formation of new compounds in processed materials. For example, to improve the ginseng function, commercial sterilization at 121 °C for 30 min was performed, and subsequently employed widely targeted metabolomics to analyze the remarkable changes of terpenoid compounds (Zhang et al., 2022). Their findings demonstrated that 11 ginsenosides, including Rh4, F4, Rg3, Rg5, and Rg6, increased after commercial sterilization, while 8 ginsenosides, including Re, Rd, Rg1, Rf1, Rb2, and Rb3, decreased (Zhang et al., 2022). These results indicate that higher temperature and higher pressure may exert differential effects on *P. ginseng* ginsenosides concentration. Another study reported that the ginsenosides Re, Rd, Rb1, Rg1, and Rg3 were detectable in ginseng roots heated to 80 °C, whereas their concentrations were reduced or became undetectable at higher temperatures (Kim et al., 2020).

In this study, a non-traditional sterilization condition (115 °C, 15 min) was used for *P. ginseng* roots processing. Compared with the commercial sterilization (121 °C, 30 min), this modified protocol significantly reduced both temperature and processing time. We observed the ginsenosides Re, Rb1, Rd, Rg2, and Rg3 in the *P. ginseng* after non-traditional sterilization treatment. Among these, ginsenoside Rg3 was absent from raw ginseng, as reported previously (Kim et al., 2000). Even more, the *P. ginseng* roots remained uncontaminated during the subsequent fermentation stage under these sterilization conditions. It indicates that the sterilization condition of 115 °C for 15 min not only generates new reference compounds but also effectively prevents environmental contamination.

Building on these findings, we used the “golden flower” fungus *A. cristatus* to ferment *P. ginseng* roots by solid fermentation following non-traditional sterilization. We observed “golden flowers” formed on the ginseng root surface and successfully produced GFCG. Similar to our results, when *A. cristatus* fermented loose-leaf dark tea, the color in both tea leaves and infusions showed distinct changes. When the color of the tea changed from dark green to brown during fermentation, the dark tea leaves’ surface became covered with golden spots at later fermentation stages (Chen et al., 2023). In a second case, in Fuzhuan brick tea (FBT) processed through unique microbial fermentation, the “golden flowers” extensively proliferated within the tea bricks, contributing to distinctive flavor and color characteristics (Zhu et al., 2017). In GFCG, *A. cristatus* appeared to transform ginsenosides Rb1 to Rd, then to Rg3, and finally to Rh2, or directly transform Rd to Rg3 and then to Rh2, or convert Re to Rg2. This fungal transformation enhanced the production of rare ginsenosides, presumably through the action of fungal de-glycosylation enzymes. Previous studies have demonstrated that Rb1, Rb2, and Rb3 could be converted to Rg3 by losing the glucose residue, while Rd could be hydrolyzed to form Rg3 and subsequently undergoes dehydration to generate Rg5 and Rk1 under commercial sterilization (Zhang et al., 2022). Another study reported that steaming red ginseng, malonyl-ginsenoside Rb1, Rb2, ginsenoside Rb1 and Rb2 decreased, while Rg3, Rd, and Rg5 increased during steaming-mediated ginsenosides transformation (Xie et al., 2012). Consistent with our results, numerous studies indicated that the extracted residues or ginsenoside mixtures could serve as substrates for incubating different fungi to produce the rare ginsenosides. For example, the fungus *Penicillium oxalicum* sp. 68 was found to transform protopanaxadiol-type ginsenosides into a series of bioactive compounds. The fungal glycosidases from this strain were utilized to transform Rb1, Rb2, Rc, and Rd to generate the compound K (Gao et al., 2013). Additionally, the fungus *Paecilomyces bainier* sp. 229 efficiently converted ginsenoside Rb1 to compound K via several ginsenoside-hydrolyzing *β*-glucosidases (Yan et al., 2010). Moreover, when red ginseng was fermented by *Monascus pilosus* KMU103, most of the ginsenosides were converted to Rh1, Rh2, and Rg3, which are de-glycosylated ginsenosides with enhanced bioavailability and pharmacological effects (Hong et al., 2011). Here, the rare ginsenosides Rg2, Rg3, and Rh2 induced by *A. cristatus* in GFCG were reported to exhibit high bioavailability and pharmacological effects, confirming the functional improvement of GFCG.

The transformation of ginseng compounds is related to fungal transcriptional and translational reprogramming. To our surprise, more than two-thirds of significantly differentially expressed genes were down-regulated in *A. cristatus* after fermentation of *P. ginseng* roots. Since *P. ginseng* is one of the important medicinal plants with plenty of bioactive compounds, the down-regulation of fungal genes might be attributed to the presence of specific chemical compounds that have a special function towards this microbe. From the plant perspective, our previous study revealed that more than 60% of *P. ginseng* genes were repressed during the interaction with gray mold fungus *B. cinerea*; however, the down-regulation of plant genes might facilitate fungal infection and disease development (Chen et al., 2022). We also observed similar results from the *B. cinerea* and *Arabidopsis thaliana* interaction, as the fungal infection down-regulated *AtWRKY33* gene expression and protein accumulation, which was the key player in plant immunity (Liu et al., 2015; Liu et al., 2017).

Microbial adaptation to environmental changes may involve transcriptomic reprogramming as indicated above. Based on GO and KEGG analysis, it is suggested that the golden flower fungus *A. cristatus* colonized the surface of *P. ginseng* roots by weakening fungal transcriptional machinery while concurrently strengthening its translational machinery, which may facilitate ginseng compounds metabolism and finally benefit microbe growth. Here, we provided a case between microbe–medicinal and edible plant interaction, which revealed the microorganisms adapt to medicinal hosts or foods by manipulating their molecular events from transcription to translation. Whether this is the general adaptive strategy employed by microbes across diverse environments remains to be investigated. In another case, it was reported that the tea field microbe *Colletotrichum camelliae* could infect the tea cultivar Longjing 43, and that fungal transcriptional reprogramming occurred at 24 h post-infection (Liu et al., 2023). A total of 5,751 SSTF genes were identified in *C. camelliae*, with approximately half of them decreasing and the other half increasing. GO analysis of these SSTF genes revealed enrichment for terms associated with transcriptional regulation, transcription factor activity, RNA polymerase II, and transmembrane transport, etc. (Liu et al., 2023). Furthermore, KEGG pathway analysis revealed several pathways associated with pathogenicity and metabolism as enriched (Liu et al., 2023). It remains unclear whether the molecular events in microbial pathogenesis during plant and microbe interaction are different from those in microbe fermentation.

Generally, the comprehensive analysis of transcriptome and metabolome can provide powerful tools to investigate potential associations between genes and metabolites involved in common biological processes and may help reveal the signaling pathway of ginseng metabolites during fungal fermentation. Previous studies have identified regulation networks based on the integrated transcriptomic and metabolomic analysis (Liu et al., 2023; Chen et al., 2022). In this study, we tried to compare the transcriptomic changes in *A. cristatus* before and after fermentation with *P. ginseng*, as well as the metabolomic changes in *P. ginseng* before and after fermentation by *A. cristatus*. However, we only observed that the ABC transporters were both significantly enriched in transcriptomic and metabolomic analysis. It is reasonable, given that transcriptomic data were generated from the golden flower fungus, while the metabolome data were obtained from ginseng roots, that the correlation between genes and metabolites was not completely from the same species. Anyway, the transcript changes would help to understand the chemical transduction mechanisms mediated by *A. cristatus*. Further study will focus on elucidating specific gene–compound relationships through combined genetic and biochemical analyses, aiming to identify which genes in *A. cristatus* are involved in the biosynthesis or biotransformation of ginseng-derived metabolites.

## 5 Conclusion

In summary, this study revealed that the golden flower fungus *A. cristatus* fermentation could strengthen the quality of *P. ginseng*. *A. cristatus* could successfully grow on the roots of *P. ginseng* and formed typical “ginseng golden flowers”. *A. cristatus* fermentation had increased ginsenosides Rg2, Rg3, and Rh2 transformation and accumulation. Untargeted metabolomic analysis of *A. cristatus* fermented *P. ginseng* revealed 446 significant differentially accumulated metabolites, with several metabolites related to pharmacological effects increasing, suggesting that this fungal fermentation improves the therapeutic effectiveness of *P. ginseng*. This study also reported the molecular characteristics of *A. cristatus* during interaction with *P. ginseng*. Around 4,850 statistically significantly differentially expressed genes in *A. cristatus* were identified, with more than 70% of them exhibiting down-regulation. While translation-related events were activated, the transcription-related events were repressed, suggesting that the fungal transcripts shifted from transcription level to translation level. The study demonstrated that *A. cristatus* fermentation confers beneficial effects on the quality of *P. ginseng*, providing valuable insights into the rational utilization of microbial fermentation technology for the enhancement of medicinal and edible plants.

## Data Availability

The data presented in the study are deposited in the NCBI repository, accession number GSE302790 (EcRS, EcCK). This data can be found here: https://www.ncbi.nlm.nih.gov/geo/query/acc.cgi?acc=GSE302790.
