# Supplementary material for: Revealing the effects of Aspergillus cristatus, golden flower fungus, fermenting on the roots of the medicinal and edible plant Panax ginseng
Source: Front Microbiol. 2026 Apr 20;17:1803757. doi: 10.3389/fmicb.2026.1803757 (PMC13136136; doi:10.3389/fmicb.2026.1803757)
Supplement: Supplementary file 6 [file Data_Sheet_6.pdf]

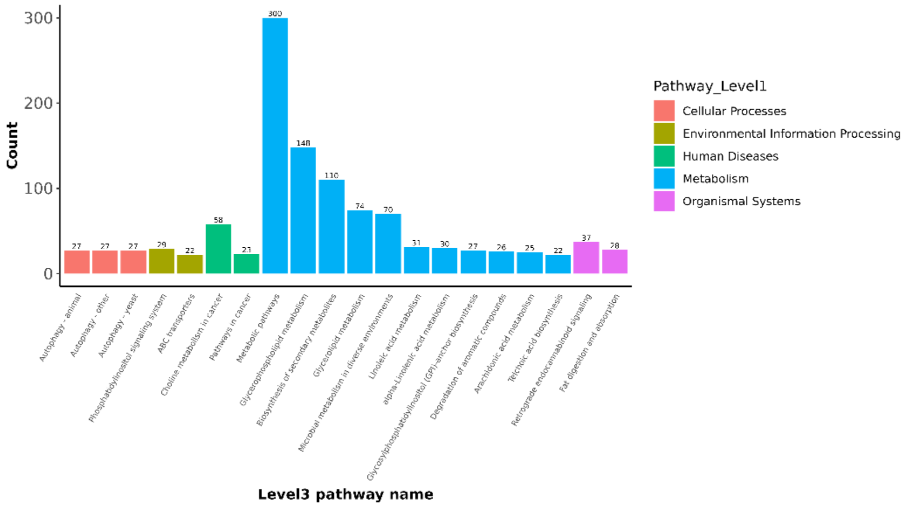

Figure S1. KEGG pathway classification. The x-axis represents level-1 terms of the KEGG pathway and the y-axis represents the number of metabolites identified.

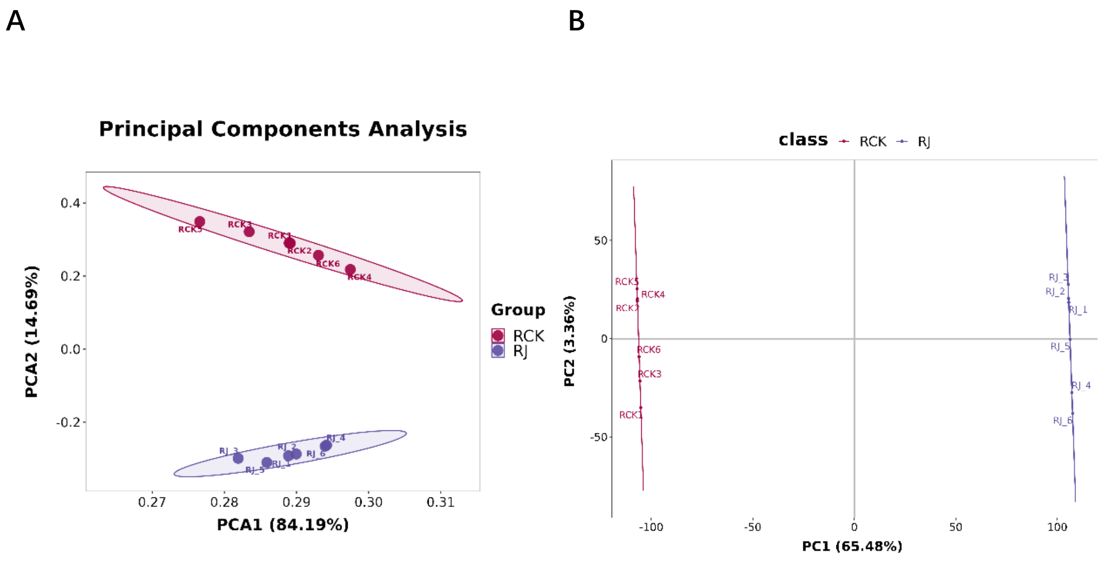

Figure S2. The principal component (A) and PLS-DA (B) analysis of the data from RCK and RJ.

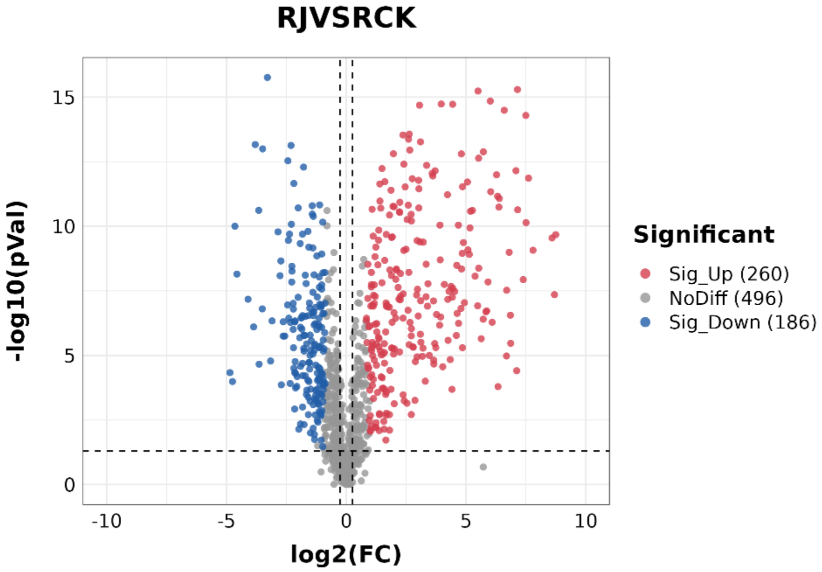

Figure S3. Identification of the differentially accumulated metabolites (DAMs) between RCK and RJ. Significance analysis of the DAMs between the treatments by Volcanoplot.

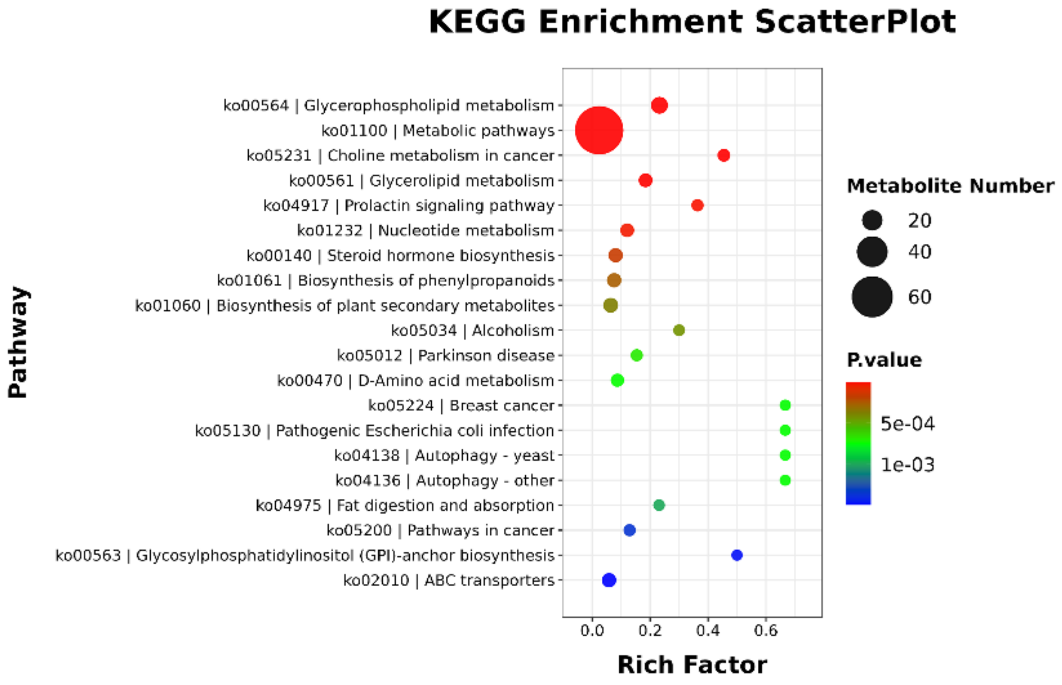

Figure S4. KEGG analysis of the significantly enriched metabolic pathways.

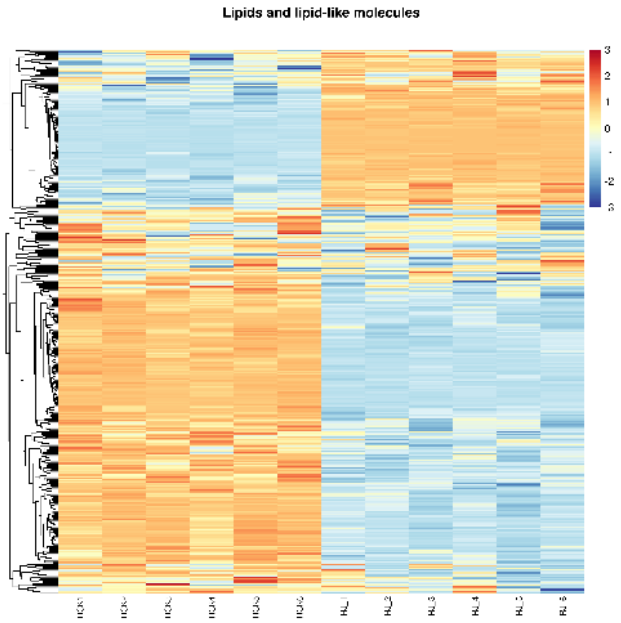

Figure S5. Heatmap analysis of the significantly enriched metabolic pathways in lipids pathway.

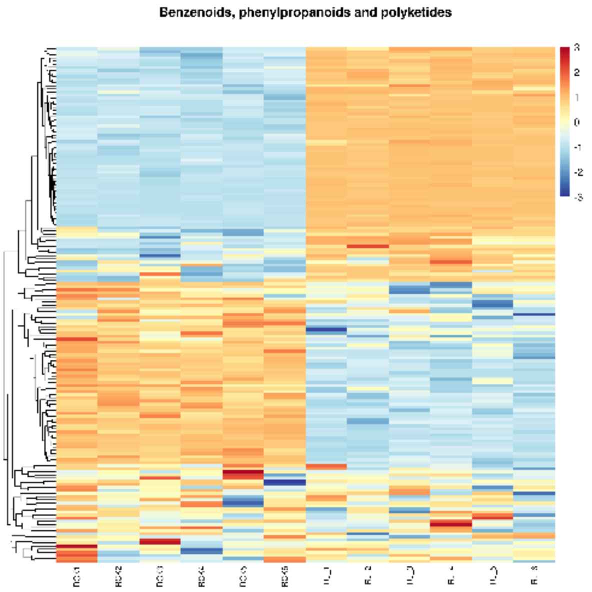

Figure S6. Heatmap analysis of the significantly enriched metabolic pathways in benzenoids, phenylpropanoids and polyketides pathway.

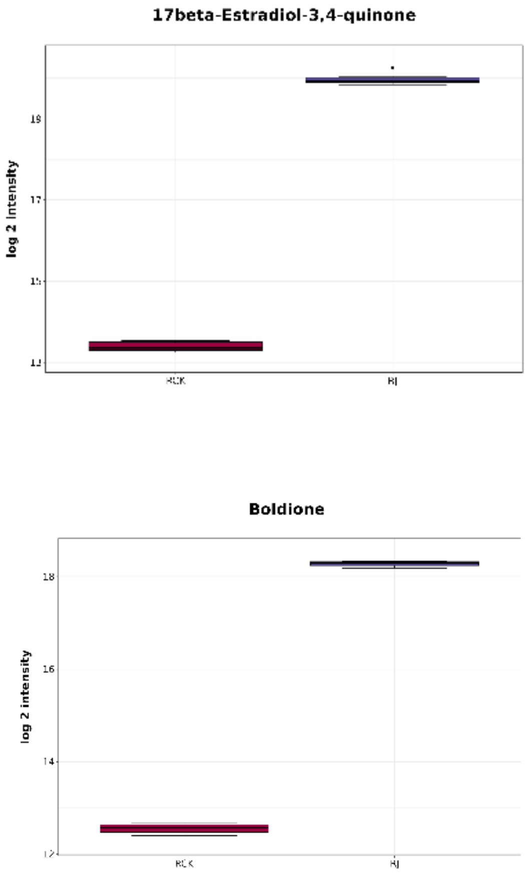

Figure S7. Differentially accumulated compounds in RCK and *A. cristatus* -treated *P. ginseng* (RJ).

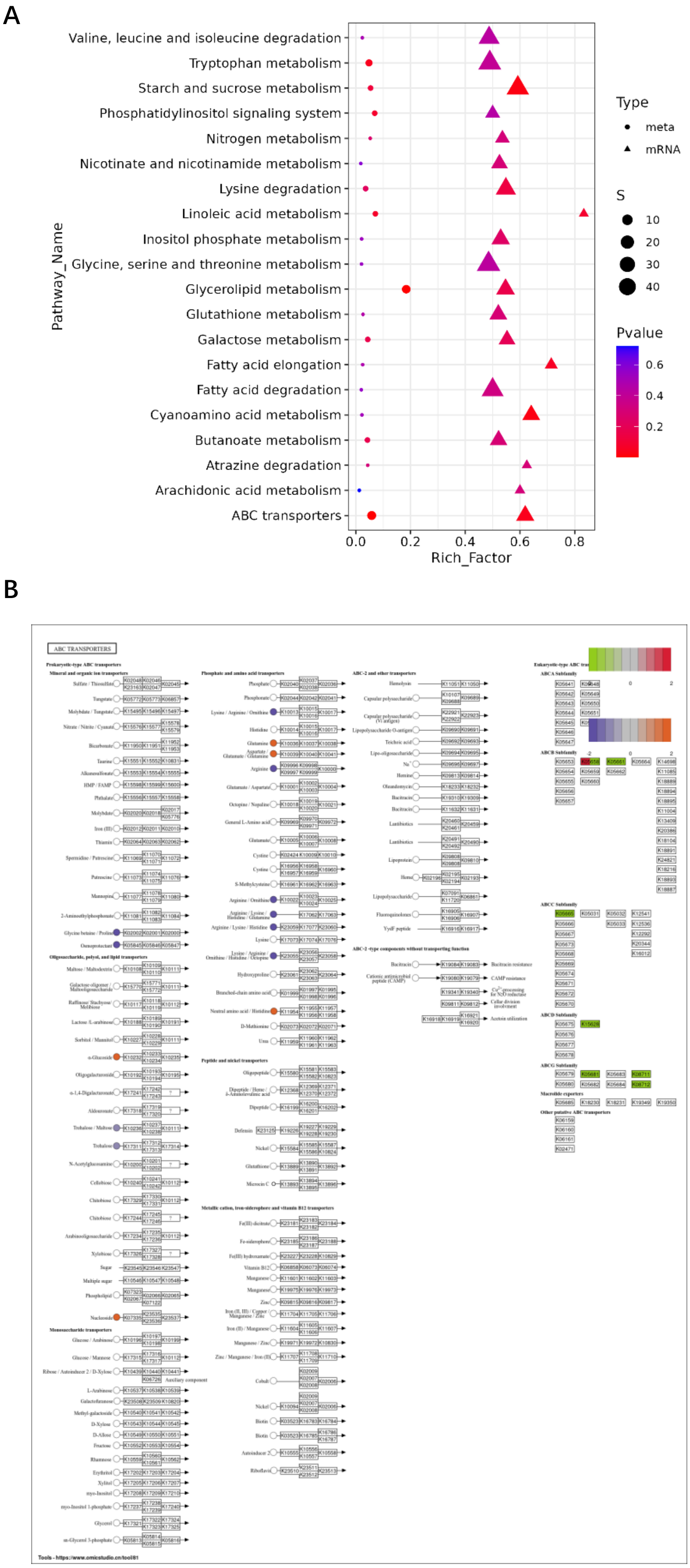

Figure S8. Identifying the co-enriched pathways in transcriptome and

metabolome. A, bubble plot of common KEGG pathways between transcripts and metabolites observed in *A. cristatus* and *P. ginseng.* In **B**, pathway map of differentially expressed genes (framed) and differentially accumulated metabolites (circled) in ABC transporter pathway.
