# Supplementary material for: Revealing the effects of Aspergillus cristatus, golden flower fungus, fermenting on the roots of the medicinal and edible plant Panax ginseng
Source: Front Microbiol. 2026 Apr 20;17:1803757. doi: 10.3389/fmicb.2026.1803757 (PMC13136136; doi:10.3389/fmicb.2026.1803757)
Supplement: Supplementary file 7 [file Table_1.docx]

| Sample | Raw Data |  | Valid Data |  | Valid Ratio(reads) | Q20% | Q30% | GC content% |
| --- | --- | --- | --- | --- | --- | --- | --- | --- |
|  | Read | Base | Read | Base |  |  |  |  |
| EcCK1 | 42221986 | 6.33G | 41111176 | 6.17G | 97.37 | 99.76 | 97.43 | 52.5 |
| EcCK2 | 44023894 | 6.60G | 42892808 | 6.43G | 97.43 | 99.79 | 97.58 | 53 |
| EcCK3 | 43009738 | 6.45G | 42177932 | 6.33G | 98.07 | 99.77 | 97.47 | 53 |
| EcRS1 | 52066688 | 7.81G | 44260666 | 6.64G | 85.01 | 99.68 | 97.73 | 53 |
| EcRS2 | 53902352 | 8.09G | 40494830 | 6.07G | 75.13 | 99.66 | 97.46 | 53 |
| EcRS3 | 50895630 | 7.63G | 40933228 | 6.14G | 80.43 | 99.69 | 97.66 | 53 |

Table S1. Total validated high-quality reads were obtained from all libraries.
